# Asymptomatic Trypanosoma cruzi Infection Identified During Blood Donation Screening

**DOI:** 10.7759/cureus.102972

**Published:** 2026-02-04

**Authors:** Jessica M Ngo, Aashna N Maknojiya, Richa Y Patel, Armando Meza

**Affiliations:** 1 Infectious Diseases, Texas Tech University Health Sciences Center El Paso Paul L. Foster School of Medicine, El Paso, USA; 2 Infectious Diseases, Texas Tech University Health Sciences Center El Paso, El Paso, USA

**Keywords:** asymptomatic infection, blood donor screening, centers for disease control and prevention (cdc), chronic chagas disease, diagnostic criteria, serologic testing, trypanosoma cruzi, us-mexico border, vector borne disease

## Abstract

Chagas disease, also known as American trypanosomiasis, is caused by the protozoan *Trypanosoma cruzi*. The global burden of Chagas disease, particularly in non-endemic regions, is increasing due to migration. In the chronic phase, which can last until treatment is administered, the majority of infected individuals remain asymptomatic. This can lead to challenges in diagnosis and increase the risk of patients developing severe, late complications. Diagnosis in chronic cases depends on serologic testing since parasitemia is low. CDC guidelines recommend confirmation with two distinct serologic assays, as screening tests alone are not sufficient for diagnosis.

We describe the case of a middle-aged woman from Panama who was found to have asymptomatic *T. cruzi* infection incidentally during routine blood donor screening. Two serologic assays per CDC guidelines confirmed the initial positive enzyme immunoassay. Limited access to first-line therapy (benznidazole) necessitated treatment with nifurtimox. The patient’s serologic titers declined following therapy, signifying a response to treatment.

This case highlights the importance of clinician familiarity with CDC diagnostic protocols, the limitations of blood donor screening as a diagnostic tool, and the treatment access barriers for Chagas disease in the United States. It also highlights the absence of standardized criteria for assessing treatment success in chronic infection. Addressing these concerns would ensure timely diagnosis, initiation of therapy, and appropriate monitoring to prevent the development of disease complications.

## Introduction

Chagas disease, or American trypanosomiasis, is caused by the protozoan *Trypanosoma cruzi* and is primarily transmitted through the feces of triatomine insects (also known as “kissing bugs”). The disease is endemic in 21 continental Latin American countries, where vector-borne transmission is most prevalent [1-3]. However, due to increasing population migration, *T. cruzi* infection is now recognized globally in non-endemic regions, including the United States, Canada, and several European, African, and Western Pacific countries [1,2].

Once introduced into the human host, trypomastigotes first invade nucleated cells, then differentiate into amastigotes, and replicate intracellularly. Upon cell lysis, trypomastigotes disseminate throughout the bloodstream to infect new cells, continuing the cycle. This process can persist for decades in the absence of treatment [3]. The acute phase is often asymptomatic or presents with mild, nonspecific symptoms. Chronically infected individuals often remain asymptomatic (indeterminate form), though some may develop severe complications, including acute myocarditis, pericardial effusion, meningoencephalitis, megacolon, or megaesophagus [1]. 

Blood donors are routinely screened for multiple diseases, including Chagas disease. According to the CDC, approximately 1 in every 27,500 blood donors tests positive for Chagas disease, making a positive screening result rare. 

Because parasitemia is low in chronic infection, diagnosis relies on serologic testing. CDC guidelines recommend confirmation of infection using two distinct serologic assays, as no single test is sufficiently reliable across all patient populations. Such tests are available through the CDC or other reference laboratories. Screening assays are not diagnostic and must be followed by confirmatory testing [4].

Early antiparasitic treatment is associated with better outcomes and reduced long-term complications [5]. Beznidazole is the preferred first-line therapy in the United States; however, its use is hindered by limited availability and regulatory barriers, often requiring a substitute such as nifurtimox, a second-line agent with different tolerability [6]. 

Unfortunately, no gold-standard test exists to confirm a cure following treatment for chronic Chagas disease. Serologic titers may decline over time, but this process is slow and variable, making post-treatment monitoring challenging [7]. 

This case describes an asymptomatic *T. cruzi *infection discovered incidentally through routine blood donation screening in a patient from an endemic region. It underscores the importance of clinician awareness of CDC diagnostic protocols, particularly in regions with high levels of immigration from endemic countries in Latin America, and the need to address barriers to treatment access. This highlights the current lack of standardized guidelines for post-treatment monitoring, emphasizing the need for defined criteria to assess therapeutic response and long-term care.

## Case presentation

A middle-aged woman with a past medical history of obstructive sleep apnea and iron deficiency anemia was referred from primary care to the infectious disease clinic to establish care following a positive serological screen for *T. cruzi* antibodies during a routine blood donation. She was asymptomatic at the time of presentation, denying any history of constipation, dysphagia, dyspnea, chest pain, peripheral edema, palpitations, or syncopal episodes. 

The patient was born in Panama and has lived in a U.S. city on the Texas-Mexico border since 1997. She reports that she frequently travels back to visit family, but does not recall any significant insect bites or illnesses associated with vector exposure. Before this, she had never attempted to donate blood and had not undergone prior cardiac imaging, such as an echocardiogram or electrocardiogram (EKG). In the past, she had undergone a chest X-ray, which came back normal, and thus no further workup was pursued at the time. An additional chest X-ray was performed to assess for possible cardiac and esophageal involvement, and results showed normal heart size, no pulmonary congestion, no focal findings, and no evidence of acute pulmonary disease. An EKG was also performed and was negative for findings suggestive of cardiac involvement from Chagas disease. Due to the patient's asymptomatic presentation and unremarkable chest X-ray and EKG, no further cardiac or GI workup was pursued.

The initial screening enzyme immunoassay (EIA, Weiner Kit) conducted by Vitalant was positive for anti-Trypanosoma cruzi antibodies in the plasma and serum samples. However, per Centers for Disease Control and Prevention (CDC) guidelines, a second confirmatory test is required for diagnosis, and testing performed by blood banks cannot be used for clinical diagnostic purposes. Confirmatory trypomastigote excreted-secreted antigens (TESA) immunoblot serologic testing was performed through LabCorp, with both tests returning positive, consistent with the indeterminate form of chronic Chagas infection. Additionally, the local Department of Public Health contacted the clinic to report that a confirmatory TESA/immunofluorescence assay (IFA) test ordered by an epidemiologist through the CDC had also returned positive.

Due to the lack of access to affordable first-line therapy, benznidazole, the patient was started on Nifurtimox 120 mg tablet orally, two tablets four times a day for three months, a second-line treatment option. Dosage was determined using 8-10 mg/kg/day, in accordance with guidelines from the 2015 Brazilian Consensus on Chagas disease.

Follow-up serologic titer demonstrated a decline from 3.2 to 2.7 over the course of one year and five months, consistent with a response to treatment. Despite recommendations for continued follow-up, the patient did not return for her final appointment and thus further assessment for end-organ damage could not be done. 

## Discussion

The diagnosis of *T. cruzi* can be distinguished by identifying the acute and chronic stages of Chagas disease. In acute infection, trypomastigotes can be observed in the patient's blood or cerebrospinal fluid. Therefore, the diagnosis of acute infection can be confirmed with thick and thin blood smears, allowing for visualization of trypomastigotes through microscopic examination [4,8]. 

However, during the chronic stage of infection, trypomastigotes are not found circulating in the blood, and serology is the most appropriate method. Given the high false-positive rate of *T. cruzi* antibody testing in Central American populations, confirmatory testing is necessary. For this reason, two or more different serologic tests to detect antibodies are required to diagnose chronic Chagas disease [8]. 

The first-line tests used for diagnosis are enzyme-linked immunosorbent assay (ELISA) based on recombinant *T. cruzi* antigen, in addition to an immunoblot (TESA) [4,8]. In the case where these tests are not in agreement, they will be repeated a second time. If the results remain contradictory, an IFA is used to determine the final result [8]. The indirect hemagglutination test is an alternative screening test performed to verify the presence of T. cruzi antibodies. This test can be particularly useful when looking at chronic disease, where parasitemia is low. The sensitivity and specificity of these tests are highly variable, as shown in Table 1.

**Table 1:** Summary of the median sensitivities and specificities for T. cruzi diagnostic testing, along with advantages and limitations. Source: [7-11]

Test	Type	Target	Advantages	Sensitivity	Specificity	Limitations/cross-reactivity
ELISA	Serology	IgG antibodies	High sensitivity/specificity; automation possible	99.0%, (95% CI: 94-100)	99.0%, (95% CI 95-100)	May cross-react with other Trypanosoma species or Leishmania; false positives in autoimmune diseases
Indirect hemagglutination test	Serology	IgG antibodies	Simple, inexpensive, widely available	75%, (95% CI: 70-80)	99%, (95% CI 96-100)	Lower sensitivity in the acute phase; cross-reactivity with Leishmania, low reproducibility
TESA immunoblot	Serology	Antibody to excreted-secreted antigens	High specificity, confirmatory	92%, (95% CI: 80–98)	100%, (95% CI: 84.6–100)	Limited availability; more expensive; cross-reactivity with Leishmania
Indirect immunofluorescence assay (IFA)	Serology	IgG antibodies	Sensitive, semi-quantitative	78%, (95% CI: 70-84)	99% (95% CI: 89-100)	Limited availability; more expensive; cross-reactivity with Trypanosoma species and Leishmania
PCR (polymerase chain reaction)	Molecular	Parasite DNA	Detects acute/chronic infection, detects low parasite load	94%, (95% CI: 82-98)	98%, (95% CI 83-100)	Cross-reactivity with Leishmania; subjective interpretation

Molecular testing can also be utilized to diagnose Chagas disease. This method can be beneficial in cases of acute Chagas disease that arise from transplant or transfusion transmission, congenital Chagas disease, and reactivated cases associated with immunosuppression. Additionally, molecular testing can be useful for monitoring laboratory exposures to *T. cruzi*. Molecular testing is conducted with two real-time PCR assays to detect *T. cruzi* DNA [12]. 

Currently, there are guidelines for blood donations in which screening tests can detect serological evidence for *T. cruzi*. If a donor has tested positive for *T. cruzi* on any screening or supplemental testing, they are no longer eligible to donate blood. However, it is important to note that these blood donor screening tests cannot be used in the clinical diagnosis of Chagas disease, and further testing is required following the methods indicated above [4]. 

In the United States, *T. cruzi *treatment is typically approached in two ways: antiparasitic therapy, which directly targets and kills the parasite, and symptomatic management, which addresses the complications and clinical manifestations of Chagas disease. Benznidazole is considered the first-line antiparasitic treatment due to its more favorable side effect profile and easy-to-follow dosing schedule. However, benznidazole is not readily available due to many factors following commercialization after FDA approval, such as high medical costs for uninsured patients, lack of an emergency delivery system, and narrow indications for use [6,13]. Therefore, the second-line treatment, nifurtimox, was used for this patient. This second-line treatment is associated with adverse effects such as nausea and weight loss, as well as central nervous system toxicity such as insomnia and peripheral neuropathy, none of which were noted by the patient [14].

Assessment of treatment efficacy and chronic Chagas disease remains challenging due to instances where parasitemia can become undetectable if the patient has a chronic infection. For example, PCR-based tests have limited sensitivities, and in cases such as these, they're not routinely used to confirm the clearance of the parasite. Instead, titers are used and IgG is monitored over time, which can serve as a marker of response to therapeutic measures [7].

Of note, this case draws attention to the role of public health in detecting and treating diseases such as Chagas. Although this patient was asymptomatic, it is known that chronic forms of Chagas disease can progress and cause irreversible cardiac and gastrointestinal complications [5]. Thus, it is crucial for early detection and treatment to be offered for the possibility of remission. Given the unique position of border cities between the United States and Mexico, it is essential for hospitals that serve patients coming from endemic regions to be familiar with the nuances of diagnosis and treatment, especially in asymptomatic patients, and to be aggressive in terms of follow-up and treatment protocols [3].

## Conclusions

Here, we present a case of asymptomatic Chagas incidentally detected upon blood donation screening. The increasing burden of Chagas in nonendemic areas highlights the need for increased clinician awareness of CDC screening guidelines, increased accessibility to first-line antiparasitic treatment, and clearly defined criteria for response to treatment. Case reports like this can assist in streamlining protocols for diagnosis, treatment selection, and post-treatment monitoring of Chagas disease in non-endemic settings. 
